# fCCAC: functional canonical correlation analysis to evaluate covariance between nucleic acid sequencing datasets

**DOI:** 10.1093/bioinformatics/btw724

**Published:** 2016-12-08

**Authors:** Pedro Madrigal

**Affiliations:** 1 Wellcome Trust-Medical Research Council Cambridge Stem Cell Institute, University of Cambridge, Cambridge, UK; 2 Wellcome Trust Sanger Institute, Wellcome Trust Genome Campus, Hixton, UK

## Abstract

**Summary:**

Computational evaluation of variability across DNA or RNA sequencing datasets is a crucial step in genomic science, as it allows both to evaluate reproducibility of biological or technical replicates, and to compare different datasets to identify their potential correlations. Here we present fCCAC, an application of functional canonical correlation analysis to assess covariance of nucleic acid sequencing datasets such as chromatin immunoprecipitation followed by deep sequencing (ChIP-seq). We show how this method differs from other measures of correlation, and exemplify how it can reveal shared covariance between histone modifications and DNA binding proteins, such as the relationship between the H3K4me3 chromatin mark and its epigenetic writers and readers.

**Availability and Implementation:**

An R/Bioconductor package is available at http://bioconductor.org/packages/fCCAC/.

**Supplementary information:**

Supplementary data are available at *Bioinformatics* online.

## 1 Introduction

Computational assessment of reproducibility across nucleic acid sequencing data is a pivotal component in genomic studies. Moreover, the ever-growing list of available datasets demands robust methods to quickly mine such resources to identify novel potential functional correlations between various genetic and epigenetic regulations. Chromatin immunoprecipitation followed by sequencing, or ChIP-seq, is a widely used method to profile histone modifications (HMs) and transcription factor (TF) binding at genome-wide scale. For each dataset, a set of peaks (regions of statistically significant read counts when compared against an IgG or input DNA controls) can be obtained (Bailey *et al.*, 2013). Reproducibility can then be evaluated by genome-wide Pearson correlation analysis, and peaks in replicates can be compared using Irreproducible Discovery Rate (IDR) analysis and/or overlap analysis (Bailey *et al.*, 2013; Li *et al.*, 2011). However, IDR was designed to find a set of reproducible peaks among different replicates of the same type, but cannot be used to compare distinct HMs or TFs datasets. Overlap analysis suffers as well from inherent statistical problems (Bardet *et al.*, 2011). The author has previously developed a methodology that, by using functional principal component analysis, revealed novel correlations between histone modifications that do not colocalize (Madrigal and Krajewski, 2015). Here, we present fCCAC, a functional canonical correlation analysis approach to allow the assesment of: (i) reproducibility of biological or technical replicates analyzing their shared covariance in higher order components; (ii) the associations between different datasets. We propose a new statistic to summarize canonical correlations that can be used instead of genome-wide (or peak based) Pearson correlation coefficient, with the advantage of using the profile of the genomic regions to study their covariance at higher orders. We assume that technical and biological replicates will share most of the variability, as will do so *bona-fide* interactions between different co-factors. Overall, fCCAC greatly facilitates the assessment of covariance in genomic applications.

## 2 Implementation

Functional data analysis is a raising field of statistics that allows moving from discrete measurements to functional approximations using an expansion in basis functions (Ramsay and Silverman, 2005). As in Madrigal and Krajewski (2015), we have used cubic splines to approximate data, which we read from genomic coverages in bigWig format. For *N* genomic regions (provided in BED format) we have two sets of curves, (*x_i_*, *y_i_*), i=1,…,N. The curves are then centered, and principal modes of variation $\rho_{\xi i}$ and $\rho_{\eta i}$ between *x_i_* and *y_i_* in terms of probe weight functions ξ and η can be estimated (Supplementary Material). The *N* pairs of probe scores represent shared variability if they correlate strongly with one another. Then, squared canonical correlations $R_{1}^{2},R_{2}^{2},\ldots ,R_{k}^{2},\;k=1,\ldots ,K$, can be calculated as in Ramsay and Silverman (2005) by constraining successive canonical probe values to be orthogonal. Values of $R_{k}^{2}$ close to 1.0 imply high covariation between the two samples (Supplementary Information). For *K* squared canonical correlations, we can compute a weighted squared correlation as $S_{K}=\sum_{k=1}^{K}R_{k}^{2}/k\leq \sum_{k=1}^{K}1/k=M$, where the weights 1/k are the *k*th harmonic number, and decrease with the order of the canonical component. Then, we can report *S_K_* as a fraction over the maximum $F(\%)=100\times (S_{K}/M)$, where *F* represents an overall measure of shared covariation. The user interacts with the main function *fccac* (examples can be found in the Supplementary Information and in the vignette of the package in Bioconductor).

## 3 Results

**Fig. 1.**
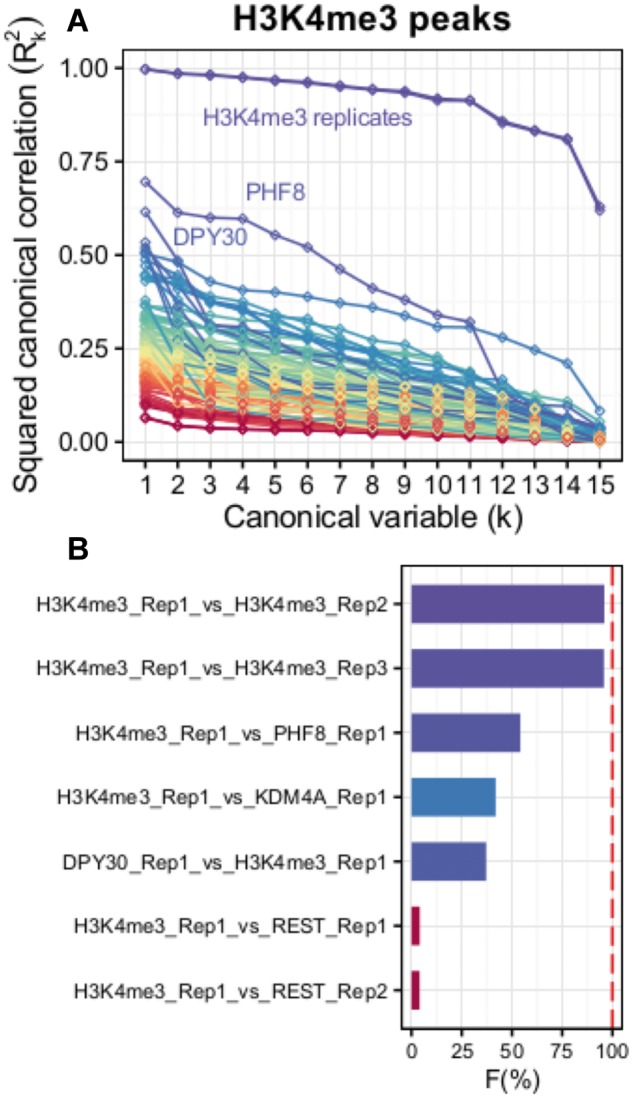
(**A**) Squared canonical correlations for H3K4me3 (Rep1) and 59 protein–DNA binding datasets (DPY30 and 58 ENCODE TFs). (**B**) First 5 and last 2 ranked interactions according to their percentage over maximum *F*. The dashed line indicates perfect covariance (Color version of this figure is available at *Bioinformatics* online.)

To exemplify the methodology we explored the correlation between the nucleosomal HM H3K4me3 and several TFs and chromatin epigenetic remodelers. For this, we focused on human embryonic stem cells (hESCs). We took advantage of recently published H3K4me3 ChIP-seq data (Bertero *et al.*, 2015), which was performed in biological triplicate from the H9 hESC line. First, we defined an aggregated list of peaks at H3K4me3 as our reference set to study replicate reproducibility (23 422 peaks). The results showed high shared covariation (*F* > 95%) for the H3K4me3 ChIP-seq triplicates, as expected (analogous analysis for H3K27me3 confirmed the irreproducibility of one of the replicates; Supplementary Material). Then, we analyzed the relationships between H3K4me3 deposition and other genomic datasets for DNA binding proteins. For this, we included ChIP-seq data for DPY30 (Bertero *et al.*, 2015), since this protein is part of the enzymatic complex responsible for the deposition of the H3K4me3 mark, as well 58 other DNA binding proteins included in the ENCODE dataset for the H1 hESC line (97 datasets) (ENCODE Project Consortium *et al.*, 2012). We found high canonical correlations between H3K4me3 and DPY30 (Fig. 1A), as expected (Bertero *et al.*, 2015). Only PHF8 (*F* = 54.2%) and KDM4A (JMJD2C) showed higher *F* value than DPY30 (*F* = 37.2%; Fig. 1B), in agreement with their known ability to bind to H3K4me3 (Feng *et al.*, 2010; Pedersen *et al.*, 2014). When we monitored all possible combinations of interactions in H3K4me3 regions, TFs BRCA1 and CHD2 showed *F* = 92% in H3K4me3, in agreement with motif analyses suggesting that they might form part of the same complex (Kheradpour and Kellis, 2014). Finally, we compared *F* to Pearson product-moment correlation coefficient. Both measures were similar between replicates of same HM or TF, but substantially differed otherwise (Supplementary Information).

## 4 Conclusion

fCCAC represents a more sophisticated approach that complements Pearson correlation of genomic coverage. This method can be used (i) to evaluate reproducibility, and flag datasets showing low canonical correlations; (ii) or to investigate covariation between genetic and epigenetic regulations, in order to infer their potential functional correlations. Overall, this method will facilitate the development of new hypothesis regarding how TFs, chromatin remodelling enzymes, histone marks, RNA binding proteins, and epitranscriptome changes can cooperatively dictate the specification of cell function and identity.

## Supplementary Material

Supplementary Data
