# Supplementary material for: fCCAC: functional canonical correlation analysis to evaluate covariance between nucleic acid sequencing datasets
Source: Bioinformatics. 2016 Dec 8;33(5):746–8. doi: 10.1093/bioinformatics/btw724 (PMC5408813; doi:10.1093/bioinformatics/btw724)
Supplement: Supplementary Data [file btw724_supp.zip › Supplementary_Information.pdf]

Supplementary Information. fCCAC: functional Canonical Correlation Analysis to evaluate Covariance between nucleic acid sequencing datasets

October 20, 2016

Contents

1 Detailed methodology **2**
1.1 Functional canonical correlation analysis 2
1.2 Details on coverage profile approximation 3

2 Availability and and installation **3**
2.1 Running time 3
2.2 Parameter recommendations 4

3 ChIP-seq datasets used **4**

4 Examples **4**
4.1 Code used to generate Figure 1 4
4.2 Reproducibility for H3K4me3 replicates (Bertero et al., 2015) . . 5
4.3 Reproducibility for H3K27me3 replicates (Bertero et al., 2015) . 5
4.4 All possible combinations of interactions in H3K4me3 peak regions 5

5 Comparison of Pearson correlation coefficient vs. functional CCA **13**
5.1 H3K4me3 replicate1 13
5.2 All possible combinations of interactions in H3K4me3 peak regions 15
5.3 H3K4me3 samples from Roadmap Epigenomics Project 15
5.4 Conclusions 15

1 Detailed methodology

1.1 Functional canonical correlation analysis

Functional data analysis is a raising field of statistics that allows moving from discrete measurements to functional approximations, e.g., using an expansion in basis functions [1, 2, 3]. Let N be the number of genomic regions in which a next generation sequencing (NGS) read coverage profile (obtained, e.g., from a ChIP-seq experiment) is observed for samples (variables) X and Y. As in [4], we have used L cubic splines $\phi_l(t)$, $l = 1, \dots, L$, (B-splines of order 4) to approximate data,

$$x_i(t) = \sum_{l=1}^{L} \alpha_{il}\phi_l(t), \tag{1}$$

$$y_i(t) = \sum_{l=1}^{L} \beta_{il}\phi_l(t), \tag{2}$$

which we read in B bins from genomic coverages in bigWig format using Genomation [5], where α and β are coefficients estimated by least squares [1]. For N genomic regions, $i = 1, \dots, N$, (peaks provided in BED format) we have therefore two sets of curves, (x_i, y_i), $i = 1, \dots, N$. The curves are then centered, and principal modes of variation $\rho_{\xi i} = \int \xi(t)x_i(t)dt$ and $\rho_{\eta i} = \int \eta(t)y_i(t)dt$ between x_i and y_i in terms of weight functions ξ and η can be estimated, under the assumption that we use the same configuration of basis functions as above. The N pairs of probe scores represent shared variability if they correlate strongly with one another. Then, squared canonical correlations ${R_1}^2, {R_2}^2, \dots, {R_k}^2$, $k = 1, \dots, K$, can be calculated as in [2] by constraining successive canonical probe values to be orthogonal,

$$ {R_k}^2(\xi_k, \eta_k) = \frac{\left[\sum_i \left(\int \xi_k(t)x_i(t)dt\right)\left(\int \eta_k(t)y_i(t)dt\right)\right]^2}{\left[\sum_i \left(\int \xi_k(t)x_i(t)dt\right)^2\right]\left[\sum_i \left(\int \eta_k(t)y_i(t)dt\right)^2\right]}, \tag{3}$$

where K should be the smallest of N, or the number of basis functions used to model x, y, or ξ, η (an error message will be printed to the user of the R/Bioconductor package `fCCAC` if the value of K is not appropriate). Values of ${R_k}^2$ close to 1.0 imply high covariance between the two samples in the genomic regions investigated. Details of funcional CCA analysis can be found in [1] and [2]. One of the advantages of functional CCA is that we can investigate canonical correlations of order $k > 1$, which can be high and/or meaningful.

For K squared canonical correlations, we can compute a weighted squared correlation S_k as

$$S_K = \sum_{k=1}^{K} \frac{{R_k}^2}{k} \leq \sum_{k=1}^{K} \frac{1}{k} = M, \tag{4}$$

where the weights $1/k$ represent the k-th harmonic number. Then, we can report S_K as a fraction over the maximum

$$F(\%) = 100 \times \left(\frac{S_K}{M}\right), \tag{5}$$

, where F is an overall measure of shared covariance. It is expected that good replicates will have F values close to 100%.

Suppl. Figure 1 shows the workflow of the methodology described above.

1.2 Details on coverage profile approximation

Genomic data in each region is first divided into B bins (parameter `nbins`) using the function `ScoreMatrixBin` in the Bioconductor package `genomation` [5]. This function then bins each window (genomic region or peak) to equal number of bins, and calculates a summary metric (maxima) for the scores of each bin, for each region, and stored in a matrix of size $N \times B$. Then, the B score summaries of each bin are approximated using a combination of B-spline basis functions as detailed in Section 1.1., by means of the function `Data2fd` in the R package `fda` [2]. This function converts an array of function values plus an array of argument values (B-spline basis) into a functional data object. Roughness penalties have been left by default (`lambda=3e-8/diff(as.numeric(range(argvals)))`), and therefore the smoothing is essentially controlled by how many basis functions are specified. Numerically, least squares approximation is performed by default using the usual textbook equations for computing the coefficients of the basis function expansions [1, 2].

2 Availability and and installation

The initial version of the package submitted to Bioconductor [6] is freely available at GitHub: http://github.com/pmb59/fCCAC/. It can be installed using `devtools`:

```
R> library("devtools")
R> devtools::install_github("pmb59/fCCAC")
```

Instructiones to install the Bioconductor package are available at: http://bioconductor.org/packages/fCCAC/. In brief, the user interacts with two functions: `fccac.R` and `heatmapfCCAC.R`. Description of parameters and functions can be found in the manual of the package. A simple example can be found in the vignette of the package, while the code used for creating Figure 1 in this manuscript can be found below. The R package `ggplot2` is used for data visualization [7].

2.1 Running time

All the examples below took between few minutes and 1h (for 101 datasets) in a computer with OS-X 2.5.GHz Intel Core i5 with 8GB RAM.

2.2 Parameter recommendations

Because heavy smoothing is suggested for functional CCA [1, 2], a low number of splines (parameter `splines`) when compared to the total length of the genomic regions is recommended. The parameter `nbins` can be low for narrow peaks (e.g., 50 for TFs and narrow chromatin marks) and increased for broad domain chromatin marks. The number of canonical correlations to compute (`ncan`) is limited by the number of splines used and the number of genomics regions to analyse (see above).

3 ChIP-seq datasets used

H3K4me3 (wild-type H9-hESCs) and H3K27me3 (H9-hESCs expressing a scrambled control shRNA and matched to the experimental sample in which a NANOG shRNA was expressed) triplicate datasets were downloaded from ArrayExpress (E-ERAD-191) and processed as in Bertero et al. (2015) [8], same as for DPY30 (E-ERAD-365). ENCODE TF ChIP-seq datasets for H1-hESC were downloaded in bigwig format from http://www.encodeproject.org/ [9]. Accessions are shown in Table 1.

Aggregate sets of reproducible peaks from Bertero et al. [8] were considered as genomic regions of interest in the analysis. Chromosome Y regions were filtered out from all samples as the cell lines have different sex, H9-hESC (female) and H1-hESC (male).

4 Examples

The purpose of fCCAC is answer questions such as "How much variation is shared between, for example, DPY30 ChIP-seq and H3K4me3 ChIP-seq in H3K4me3 domains?" or, "how much variation is shared between BRCA1 ChIP-seq and CTCF ChIP-seq in H3K4me3 domains?". The examples below will address these questions in datasets of human embryonic stem cells (hESCs) using fCCAC.

All necessary files, except bigWig files, can be found in Suppl. Material.

4.1 Code used to generate Figure 1

The the commands used for analyzing the data and generating Figure 1 are:

```
R> library("fCCAC")
R> encode <- read.table("ENCODE.txt", head=F)
R> encode <- encode[order(encode$V2),]
R> head(encode)
V1 V2
ATF2_ENCFF000OMJ.bigWig ATF2
ATF2_ENCFF000OMK.bigWig ATF2
ATF3_ENCFF000OMQ.bigWig ATF3
ATF3_ENCFF000OMS.bigWig ATF3
BACH1_ENCFF000WQO.bigWig BACH1
```

```
BCL11A_ENCFF000OMX.bigWig BCL11A
fc <- fccac(bar=c(5,2), main="H3K4me3 peaks",tf="H3K4me3_Rep1",
peaks="noY_chr_merged_ACT_K4.bed", bigwigs= as.character(encode$V1)
, labels= as.character(encode$V2) , splines=15, nbins=100, ncan=15)
```

4.2 Reproducibility for H3K4me3 replicates (Bertero et al., 2015)

```
R> library("fCCAC")
R> bigwigs <- c("H3K4me3_1.bw", "H3K4me3_2.bw", "H3K4me3_3.bw" )
R> labels <- c( "H3K4me3", "H3K4me3","H3K4me3" )
R> fc <- fccac(bar=NULL, main="H3K4me3 peaks", peaks="noY_chr_merged_ACT_K4.bed",
bigwigs=bigwigs, labels=labels , splines=15, nbins=100, ncan=15)
```

The output produced is shown in Suppl. Figure 2.

4.3 Reproducibility for H3K27me3 replicates (Bertero et al., 2015)

```
R> library("fCCAC")
R> bigwigs <- c("H3K27me3_1.bw", "H3K27me3_2.bw", "H3K27me3_3.bw" )

R> labels <- c( "H3K27me3", "H3K27me3","H3K27me3" )
R> fc <- fccac(bar=NULL, main="H3K27me3 peaks", peaks="noY_chr_merged_ScrN_K27.bed",
bigwigs=bigwigs, labels=labels, splines=15, nbins=100, ncan=15)
```

The output produced is shown in Suppl. Figure 3.

4.4 All possible combinations of interactions in H3K4me3 peak regions

```
R> library("fCCAC")
R> fc <- fccac(bar=c(10,10), main="H3K4me3 peaks", peaks="noY_chr_merged_ACT_K4.bed",
bigwigs= as.character(encode$V1) , labels= as.character(encode$V2)
, splines=15, nbins=100, ncan=15)
```

The output produced is shown in Suppl. Figure 4. A screenshot with an example of the covariance between BRCA1 and CDH2 in H3K4me3 peaks is shown in Suppl. Figure 5.

When all pairwirse comparisons are calculated, like in the example above (`tf=c()`), the Bioconductor package fCCAC includes an additional function that performs unsupervised hierarchical clustering (Euclidean distance, complete method) of the F values, simply by doing:

```
R> heatmapfCCAC(fc)
```

The heatmap produced is shown in Suppl. Figure 6. Not suprisingly we can see a cluster of CTCF-RAD21 with high F values [10], but also other clusters

with USF1/2-ATF3 and E2F6-MAX (MAX is a member of the E2F6 complex in ES cells [11]).

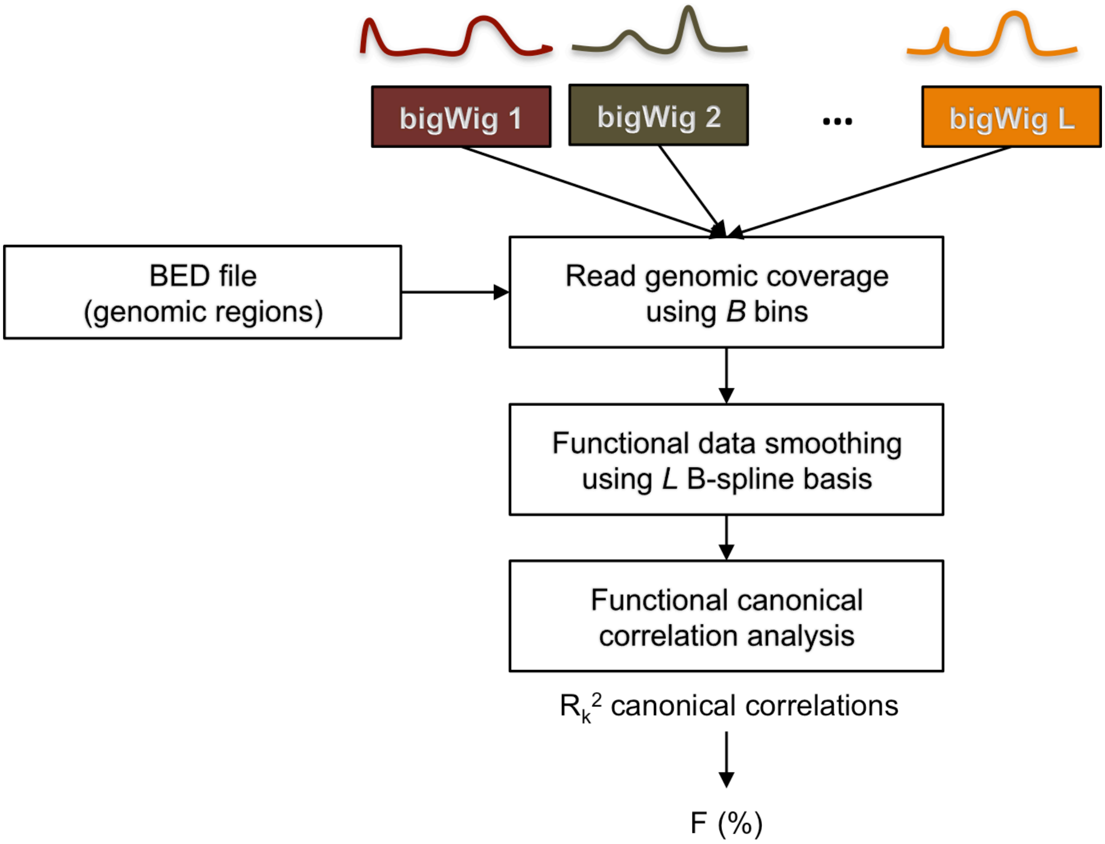

Supp. Figure 1. Workflow of the R/Bioconductor package fCCAC.

Sample	Target	Accession	Sample	Target	Accession
1	BCL11A	ENCFF000OMX	50	NANOG	ENCFF000OQC
2	CHD2	ENCFF000WRU	51	GABPA	ENCFF000OOW
3	ATF2	ENCFF000OMJ	52	EGR1	ENCFF000OOE
4	ATF2	ENCFF000OMK	53	EGR1	ENCFF000OOG
5	ZNF143	ENCFF000WWG	54	RAD21	ENCFF000ORX
6	USF1	ENCFF000OWB	55	RAD21	ENCFF000ORZ
7	USF1	ENCFF000OWC	56	TAF1	ENCFF000OUV
8	GTF2F1	ENCFF000WSU	57	TAF1	ENCFF000OUW
9	BACH1	ENCFF000WQO	58	MXI1	ENCFF000WUG
10	SP1	ENCFF000OTP	59	KDM5B	ENCFF000AZK
11	SP1	ENCFF000OTQ	60	POU5F1	ENCFF000ORO
12	E2F6	ENCFF000ONX	61	POU5F1	ENCFF000ORQ
13	E2F6	ENCFF000ONY	62	YY1	ENCFF000OWJ
14	CREB1	ENCFF000ONG	63	YY1	ENCFF000OWK
15	CREB1	ENCFF000ONJ	64	TAF7	ENCFF000OVD
16	USF2	ENCFF000WVY	65	TAF7	ENCFF000OVF
17	TBP	ENCFF000WVT	66	MYC	ENCFF000WSI
18	SUZ12	ENCFF000WVM	67	RAD21	ENCFF000WUV
19	TCF12	ENCFF000OVL	68	SIN3A	ENCFF000OSZ
20	TCF12	ENCFF000OVM	69	SIN3A	ENCFF000OTB
21	SP4	ENCFF000OUE	70	TEAD4	ENCFF000OVT
22	SP4	ENCFF000OUF	71	TEAD4	ENCFF000OVU
23	SIX5	ENCFF000OTH	72	EP300	ENCFF000OQR
24	SIX5	ENCFF000OTI	73	EP300	ENCFF000OQS
25	CTBP2	ENCFF000WSM	74	RXRA	ENCFF000OSQ
26	SP2	ENCFF000OTY	75	RXRA	ENCFF000OSR
27	SP2	ENCFF000OTZ	76	SRF	ENCFF000OUN
28	EZH2	ENCFF000AVT	77	SRF	ENCFF000OUP
29	KDM4A	ENCFF000AYY	78	ZNF274	ENCFF000WWT
30	SIRT6	ENCFF000AZZ	79	CHD1	ENCFF000WRN
31	EP300	ENCFF000AZC	80	ATF3	ENCFF000OMQ
32	RFX5	ENCFF000WUZ	81	ATF3	ENCFF000OMS
33	JUN	ENCFF000WSA	82	CTCF	ENCFF000ONO
34	MAFK	ENCFF000WTS	83	CTCF	ENCFF000ONQ
35	CEBPB	ENCFF000WRF	84	BCL11A	ENCFF000ONB
36	PHF8	ENCFF000AZH	85	FOSL1	ENCFF000OON
37	RBBP5	ENCFF000AZQ	86	FOSL1	ENCFF000OOP
38	SAP30	ENCFF000AZU	87	SIN3A	ENCFF000WVI
39	HDAC6	ENCFF000AYO	88	CTCF	ENCFF000RSB
40	MYC	ENCFF000RRW	89	CTCF	ENCFF000RSD
41	MYC	ENCFF000RRZ	90	CHD7	ENCFF000AVE
42	KDM5A	ENCFF000AYU	91	JUND	ENCFF000WTJ
43	CHD1	ENCFF000AUX	92	MAX	ENCFF000OPT
44	SUZ12	ENCFF000BAD	93	MAX	ENCFF000OPU
45	HDAC2	ENCFF000AYL	94	BRCA1	ENCFF000WQX
46	CTCF	ENCFF000AVN	95	JUND	ENCFF000OPM
47	REST	ENCFF000OQJ	96	JUND	ENCFF000OPN
48	REST	ENCFF000OQK	97	NRF1	ENCFF000WUN
49	NANOG	ENCFF000OQB			

Table 1: ENCODE accessions for ChIP-seq datasets used.

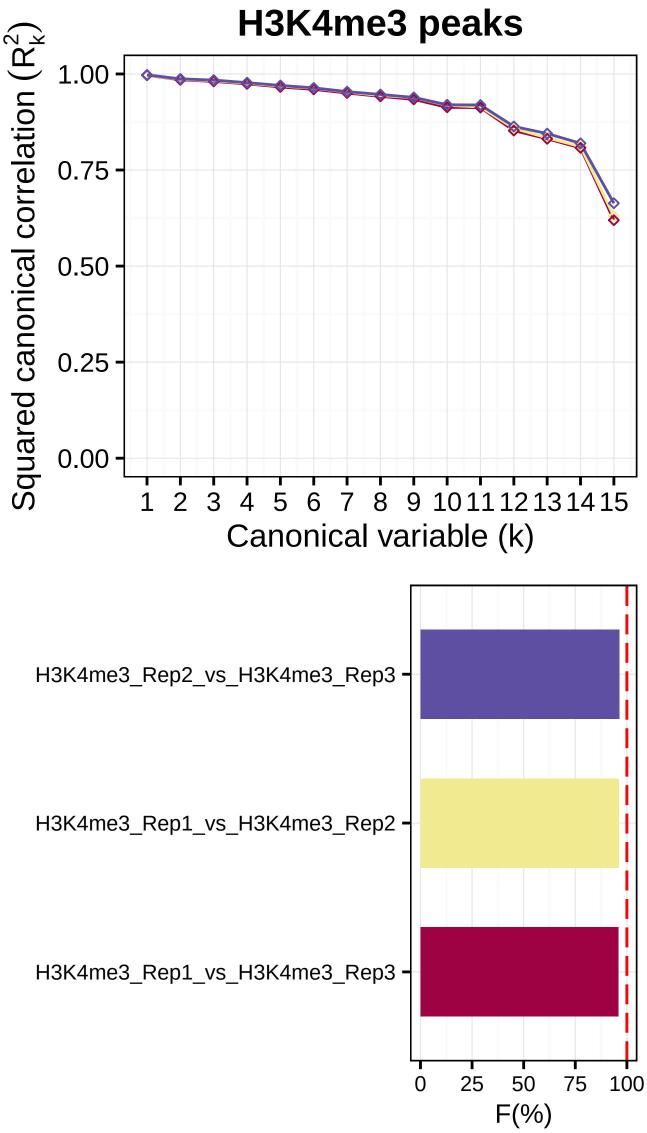

Supp. Figure 2. Assesment of covariance in H3K4me3 triplicates using functional canonical correlation analysis implemented in `fCCAC`. In the original study [8], peaks in the replicates presented Pearson correlation coefficient $r > 0.98$ for H3K4me3. Similar results were obtained for r computed genome-wide.

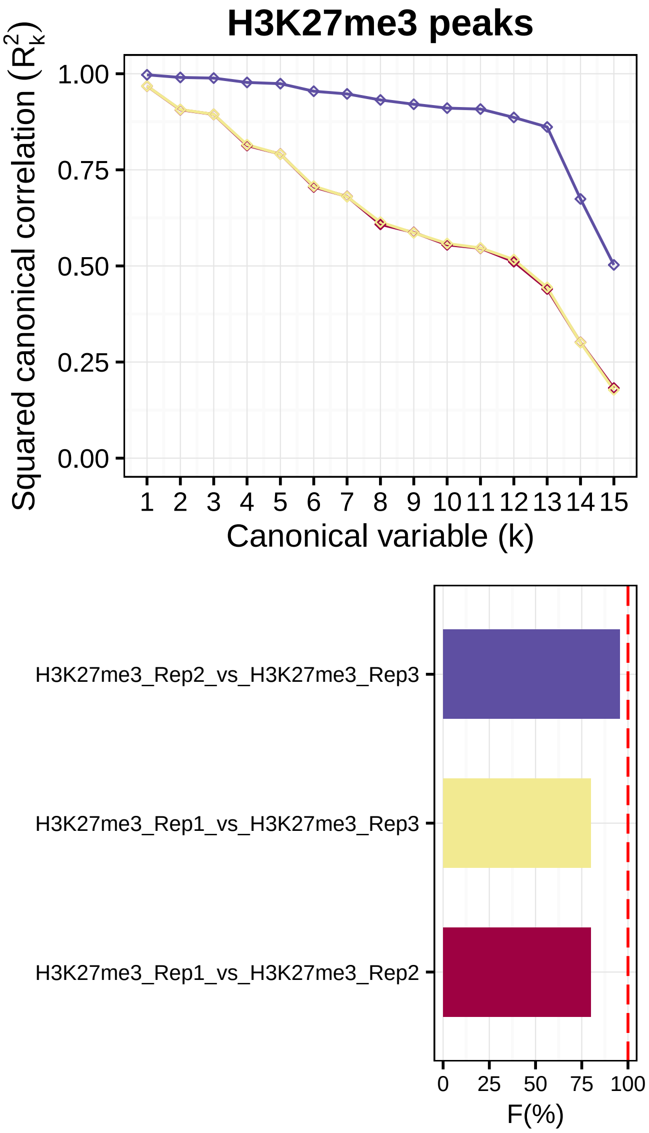

Supp. Figure 3. Assesment of covariance in H3K27me3 triplicates using functional canonical correlation analysis implemented in `fCCAC`. Only replicates Rep2 and Rep3 present a F value close to the theoretical maximum for perfect covariance after accounting the contribution of the individual components $k = 1, 2, \ldots, 15$. In the original study [8], peaks in the replicates Rep2 and Rep3 presented Pearson correlation coefficient $r > 0.95$, while $r < 0.74$ when both were compared to Rep1, which was discarded. Similar results were obtained for r computed genome-wide.

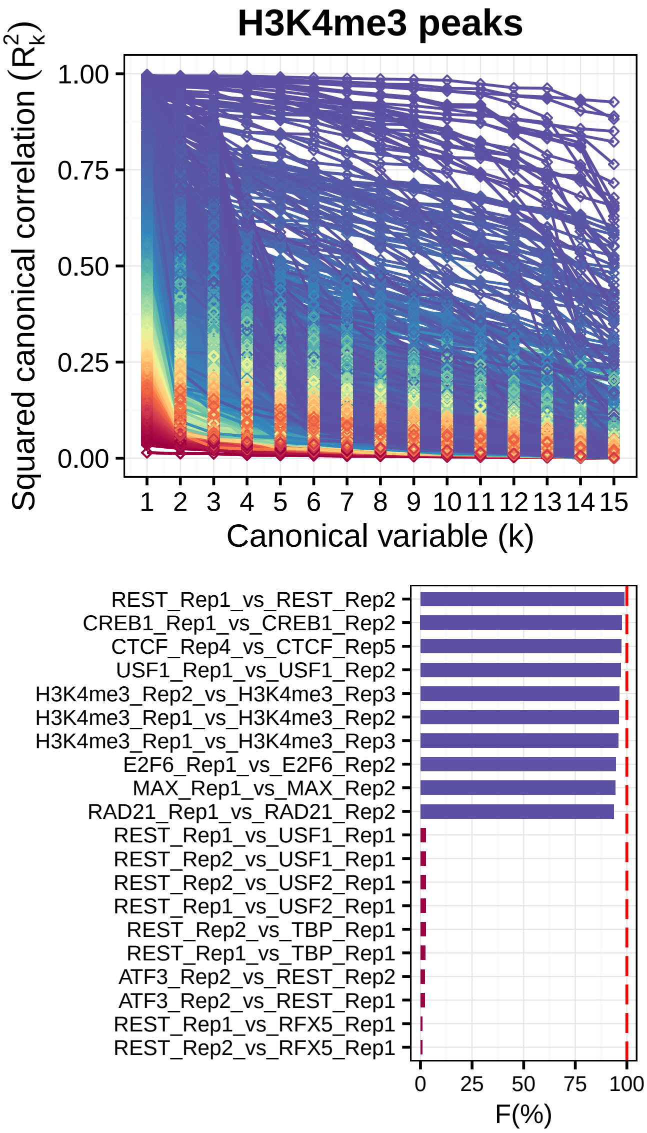

Supp. Figure 4. Top. Squared canonical correlations for all interactions between datasets (H3K4me3, DPY30 and 58 ENCODE TFs in hESCs). Spectral colormap based in the value for $k = 1$. Bottom: First 10 and last 10 ranked interactions according to their percentage over maximum F. The red dashed line indicates perfect covariance. The results for first 10 interactions reveal high covariance in highly reproducible biological replicates, while on the contrary REST and RFX5 present very low shared variability in the genomic regions investigated (see supplementary table for a list of all correlations).

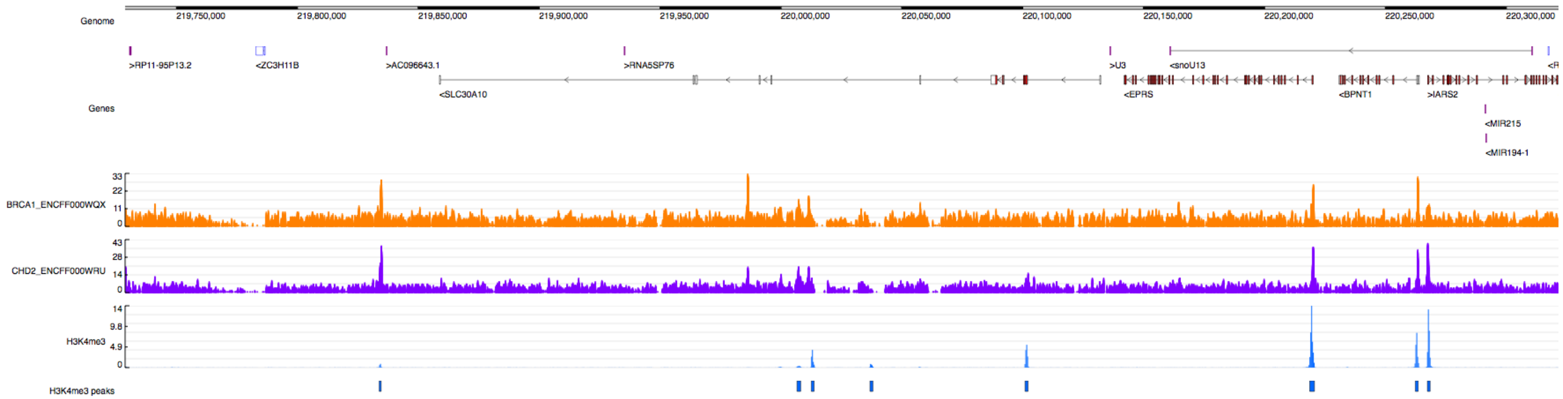

Supp. Figure 5. Dalliance screenshot (http://www.biodalliance.org/human37.html) showing ChIP-seq datasets for BRCA1 and CDH2 (ENCODE), as well as H3K4me3 signal and peaks (blue color) from Bertero et al. [8]. High covariance between the two TFs is observed in H3K4me3 regions, suggesting a previously unappreciated functional association between these proteins and H3K4me3 nucleosomal histone modification in human embryonic stem cells.

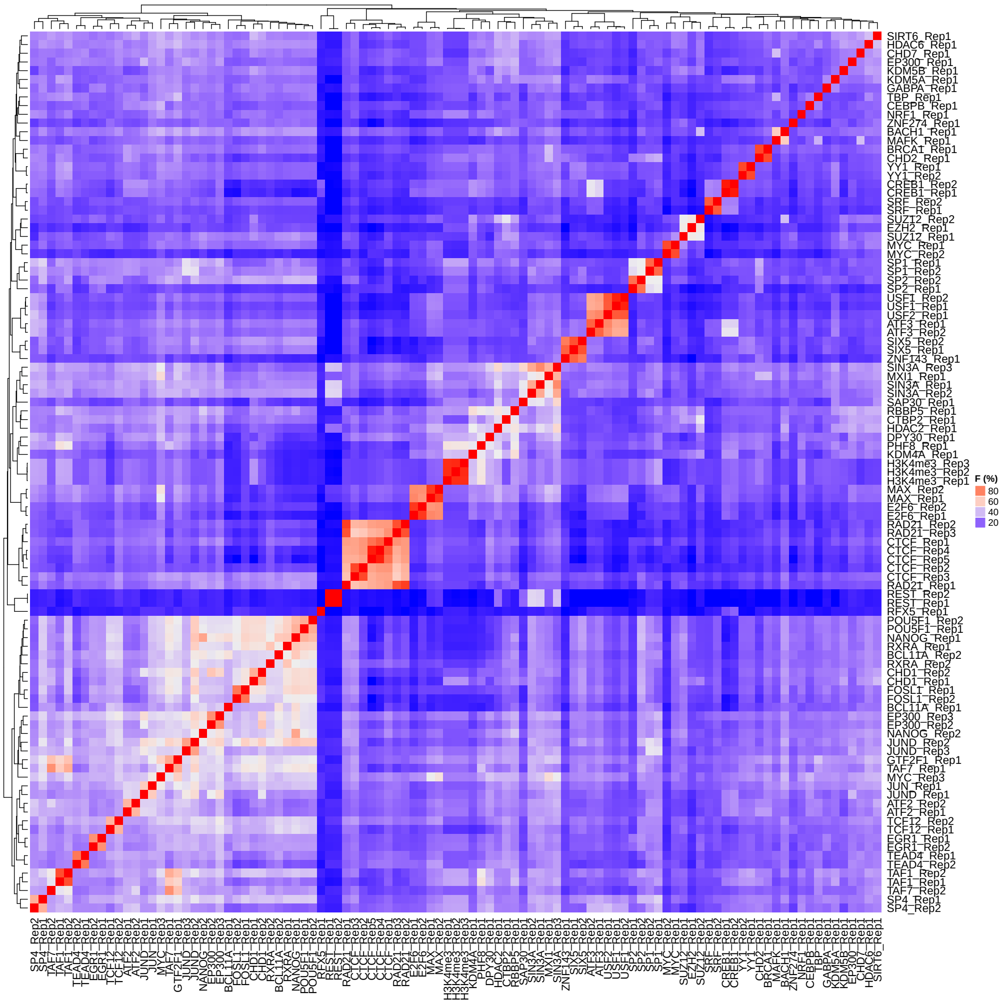

Supp. Figure 6. Heatmap for F values for all possible combinations of interactions in H3K4me3 peak regions.

5 Comparison of Pearson correlation coefficient vs. functional CCA

We computed the Pearson product moment correlation coefficient (r) using the UCSC tool `bigWigCorrelate` with the option `-restrict` so that r is only computed in H3K4me3 regions used in the fCCAC examples shown in previous sections. As before, spectral colormap order is based on the value in $k = 1$.

5.1 H3K4me3 replicate1

This analysis corresponds to data shown in Figure 1 in the main text. First, we see that replicates for H3K4me3 present both very high Pearson's r and F values.

However, despite DPY30 having the highest Pearson r with H3K4me3_Rep1(except with other two H3K4me3 replicates), PHF8 and KDM4A present higher F values (Supp. Figure 7). This is because first functional canonical correlation was higher for PHF8, and also because functional canonical correlations of order $k > 2$ were again higher for PHF8 and KDM4A (Figure 1; Supp. Figure 8). In addition, SP1 and SP4 have low r but moderate levels of F (for SP1, $r = 0.03$; $F = 26.95\%$ and $F = 34.92\%$ for H3K4me3_Rep1_vs_SP1_Rep1 and H3K4me3_Rep1_vs_SP1_Rep2, respectively). We can find in the literature association between H3K4me3 and SP1 in hESCs [12], and between SP1 and SP4 as good predictors of H3K4me3 in H1 ES cells [13]. This suggests that fCCAC can find known associations that otherwise are unrecognized if measuring Pearson's correlation coefficient r.

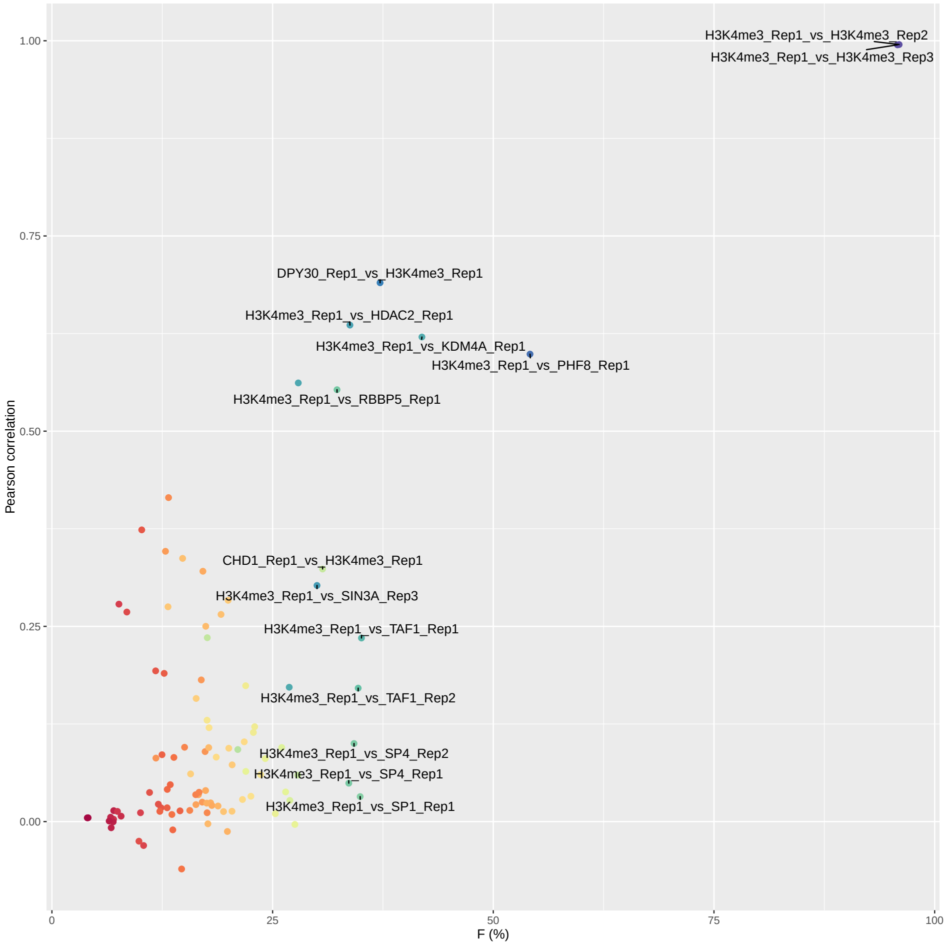

Supp. Figure 7. Pearson correlation coefficient versus F values for H3K4me3 (Rep1). Only comparisons with $F > 30\%$ have been labelled in the plot.

5.2 All possible combinations of interactions in H3K4me3 peak regions

When we monitor all possible combinations of interactions in H3K4me3 regions, and compare Pearson's r with F values, we observe highest values in both correspond to biological replicates (Supp. Figure 9). Comparisons with lower F values revealed TF REST and other factors in H3K4me3 domains, suggesting that REST binding does not share variation with the other factors analysed (Supp. Figure 9), all of them having low levels of Pearson's r.

However, we observed also strong differences between the two measures of correlation. On the one hand, pairwise comparisons with very high r and low F (Supp. Figure 10), and on the other hand low r and intermediate-high F values (Supp. Figure 11). The latter includes moderate-high F values for chromatin remodelling factor CHD1 and pluripotency factors NANOG and POU5F1 (OCT4) in H3K4me3 domains. This confirms the known interaction between CHD1 and H3K4me3 in embryonic stem cells, and its importance in the pluripotency network [14, 15].

Hierarchical clustering for the Pearson correlation coeficients was performed as before for F values in Supp. Figure 6. The differences with respect to F are also reflected in a different clustering (Supp. Figure 12). For instance, BRCA1 and CHD2 do not form part of the same cluster now, same as for USF1 and ATF3. However, clusters of MAX-E2F6 and CTCF-RAD21 are also observed using Pearson's r (Supp. Figure 12).

5.3 H3K4me3 samples from Roadmap Epigenomics Project

We then run fCCAC on H3K4me3 human datasets (same parameters as above) for different ES and iPS cell lines of the Roadmap Epigenomics Consortium [16], and included a H3K36me3 sample in brain as an outlier control. Data used is shown in Table 2. -log10(p-value) signal tracks were downloaded in bigWig format from:
http://egg2.wustl.edu/roadmap/web_portal/processed_data.html. When we compared same histone modification (different cell lines) in the peak regions for H3K4me3 as before (peak data from H9s H3K4me3, Bertero et al. [8]) we did not see strong differences with Pearson's r (Supp. Figure 13). As expected, clustering of F values showed the control sample as an outlier with respect the H3K4me3 ChIP-seq (Supp. Figure 13).

5.4 Conclusions

When we compared ChIP-seq biological replicates for the same factor (HM or TF) of interest, or same factor across different laboratories the results of Pearson correlation coefficients and fCCAC were very similar. However, when finding associations between different factors both approaches were dissimilar. This is not surprising, as both methodologies pursue different objectives. The standard Pearson product moment correlation coefficient r measures the extent to which two variables are related, while funcional CCA identifies dominant modes of variation that are correlated between two variables. Therefore, one can conclude that one approach would complement the other, but fCCAC would be most advantageous when comparing data from different TFs or histone marks - rather

Epigenome ID	Cell type	Assay	File
E001	ES-I3	H3K4me3	E001-H3K4me3.pval.signal.bigwig
E002	ES-WA7	H3K4me3	E002-H3K4me3.pval.signal.bigwig
E003	H1	H3K4me3	E003-H3K4me3.pval.signal.bigwig
E008	H9	H3K4me3	E008-H3K4me3.pval.signal.bigwig
E014	HUES48	H3K4me3	E014-H3K4me3.pval.signal.bigwig
E015	HUES6	H3K4me3	E015-H3K4me3.pval.signal.bigwig
E016	HUES64	H3K4me3	E016-H3K4me3.pval.signal.bigwig
E018	iPS-15b	H3K4me3	E018-H3K4me3.pval.signal.bigwig
E019	iPS-18	H3K4me3	E019-H3K4me3.pval.signal.bigwig
E020	iPS-20b	H3K4me3	E020-H3K4me3.pval.signal.bigwig
E021	iPS DF 69	H3K4me3	E021-H3K4me3.pval.signal.bigwig
E022	iPS DF 19.11	H3K4me3	E022-H3K4me3.pval.signal.bigwig
E024	ES-UCSF4	H3K4me3	E024-H3K4me3.pval.signal.bigwig
E072	Brain Inf. Temp. Lobe	H3K36me3	E072-H3K36me3.pval.signal.bigwig

Table 2: Roadmap Epigenomics Project ChIP-seq data used in section 5.3.

than when assesing reproducibility of biological or technical replicates. Furthermore, it is important to remind that information in higher order components obtained from high-throughput sequencing data also influences gene expression [4, 17, 18], and these cannot be studied traditional measures of correlation.

References

[1] Ramsay, J.O., Silverman, B.W. (2005) *Functional Data Analysis.* Springer-Verlag, New York.

[2] Ramsay, J.O. *et al.* (2009) *Functional Data Analysis with R and MATLAB.* Springer-Verlag, New York.

[3] Ullah, S., and Finch, C.F. (2013) Applications of functional data analysis: A systematic review. *BMC Med Res Methodol* , **13**, 43.

[4] Madrigal, P., and Krajewski, P. (2015) Uncovering correlated variability in epigenomic datasets using the Karhunen-Loeve transform. *BioData Min.*, **8**, 20.

[5] Akalin, A. *et al.* (2015) Genomation: a toolkit to summarize, annotate and visualize genomic intervals. *Bioinformatics*, **31**, 1127-1129.

[6] Gentleman, R.C. *et al.* (2004) Bioconductor: open software development for computational biology and bioinformatics. *Genome Biol.*, **5**, R80.

[7] Wickham, H. (2009) *ggplot2: Elegant Graphics for Data Analysis.* Springer-Verlag, New York.

[8] Bertero, A. *et al.* (2015) Activin/nodal signaling and NANOG orchestrate human embryonic stem cell fate decisions by controlling the H3K4me3 chromatin mark. *Genes Dev.*, **29**, 702-717.

[9] ENCODE Project Consortium *et al.* (2012) An integrated encyclopedia of DNA elements in the human genome. *Nature*, **489**, 57-74.

[10] Ong, C., and Corces, V.G. (2014) CTCF: an architectural protein bridging genome topology and function. *Nature Rev. Genet*, **15**, 234–246.

[11] Maeda, I. *et al.* (2013) Max is a repressor of germ cell-related gene expression in mouse embryonic stem cells. *Nat Commun.*, **4**, 1754.

[12] Kelley, D., and Rinn, J. (2012) Transposable elements reveal a stem cell-specific class of long noncoding RNAs. *Genome Biol.*, **13**, R107.

[13] Benveniste, D. *et al.* (2014) Transcription factor binding predicts histone modifications in human cell lines. *PNAS*, **111**, 13367-13372.

[14] Gaspar-Maia, A. *et al.* (2009) Chd1 regulates open chromatin and pluripotency of embryonic stem cells. *Nature*, **460**, 863-868.

[15] Harikumar, A., and Meshorer, E. (2015) Chromatin remodeling and bivalent histone modifications in embryonic stem cells. *EMBO Rep.*, **16**, 1609-1619.

[16] Roadmap Epigenomics Consortium. *et al.* (2015) Integrative analysis of 111 reference human epigenomes. *Nature*, **518**, 317-330.

[17] Schweikert, G. *et al.* (2013) MMDiff: quantitative testing for shape changes in ChIP-Seq data sets. *BMC Genomics*, **14**, 826.

[18] Cremona, M.A. *et al.* (2015) Peak shape clustering reveals biological insights. *BMC Bioinformatics*, **16**, 349.

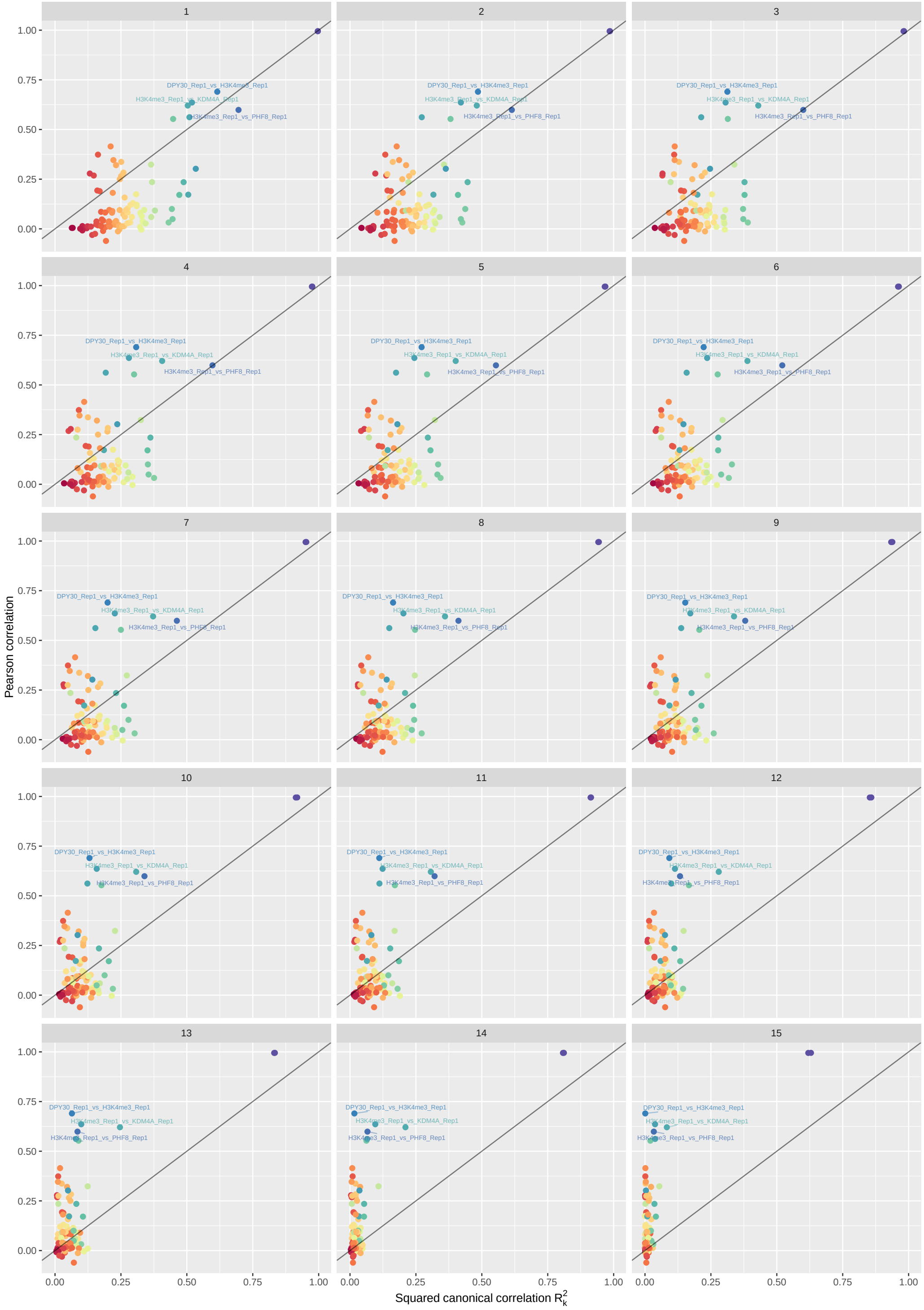

Supp. Figure 8. Pearson correlation coefficient r versus squared canonical correlations of order k for H3K4me3 (Rep1). Only comparisons with DP30, PHF8 and KDM4A have been labelled in the plot.

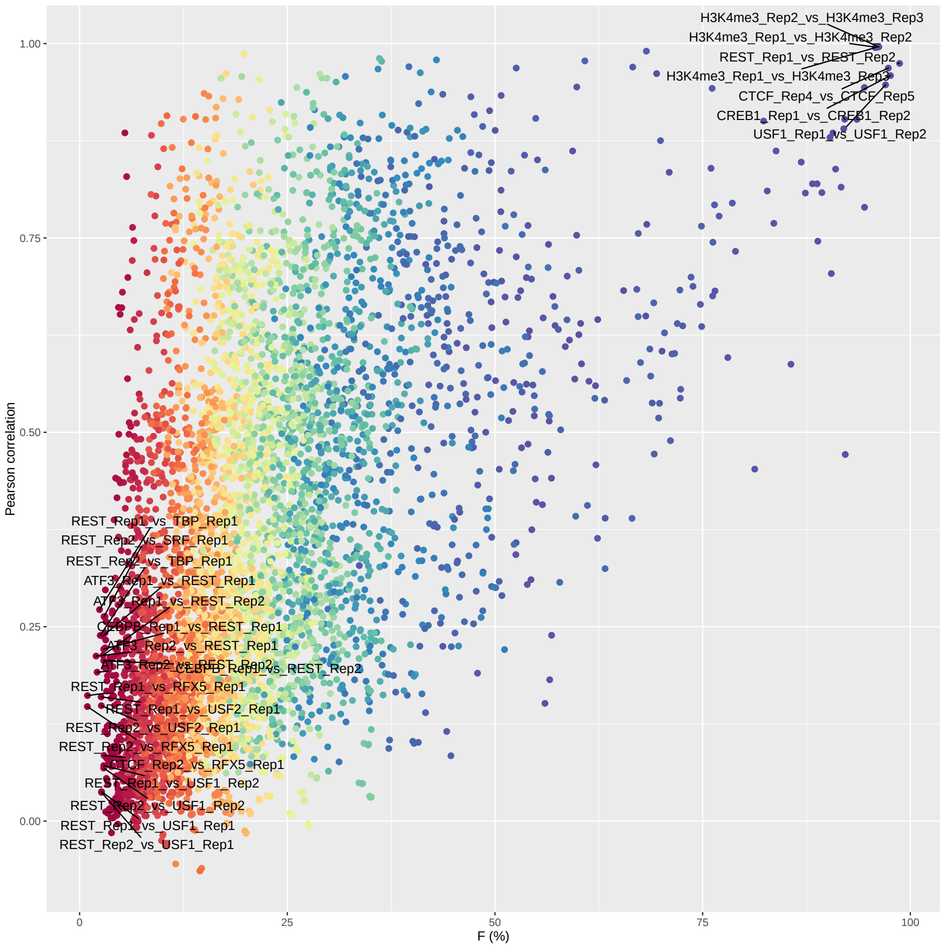

Supp. Figure 9. Pearson correlation coefficient versus F values for all combinations of interactions in H3K4me3 regions. Only comparisons with 3% > F > 95% have been labelled in the plot.

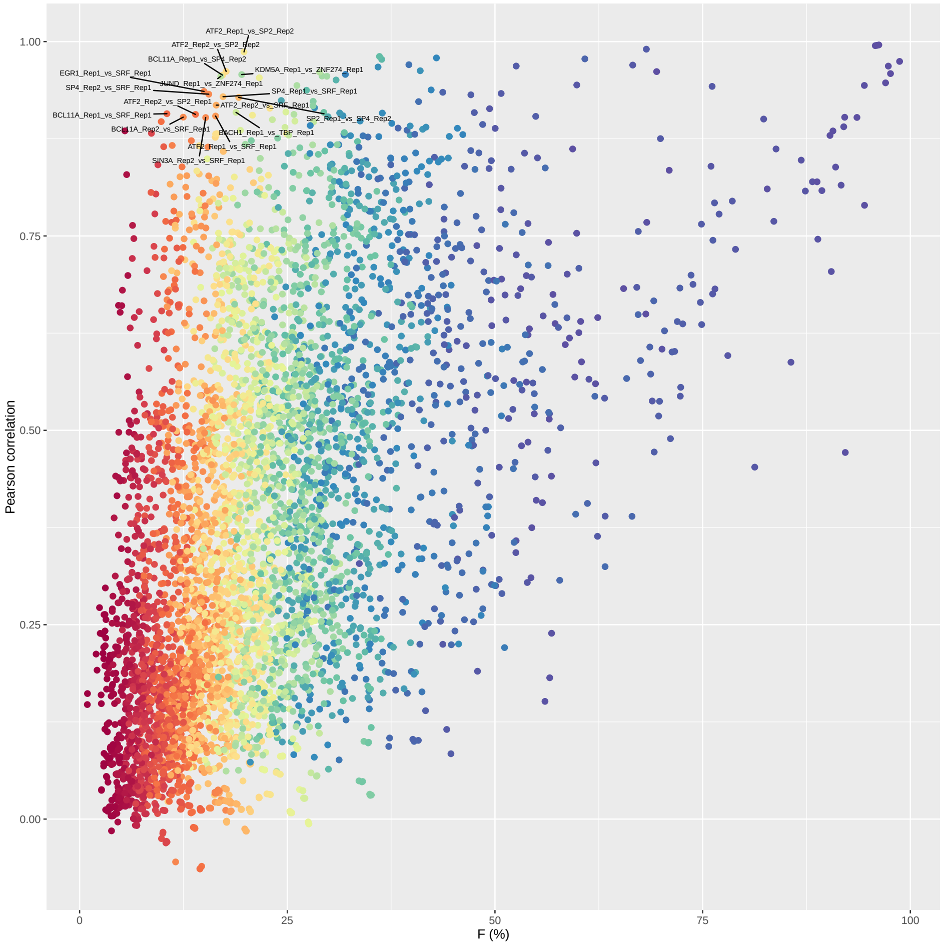

Supp. Figure 10. Pearson correlation coefficient versus F values for all combinations of interactions in H3K4me3 regions. Only comparisons with $F < 20\%$ and Pearson's $r > 0.9$ have been labelled in the plot.

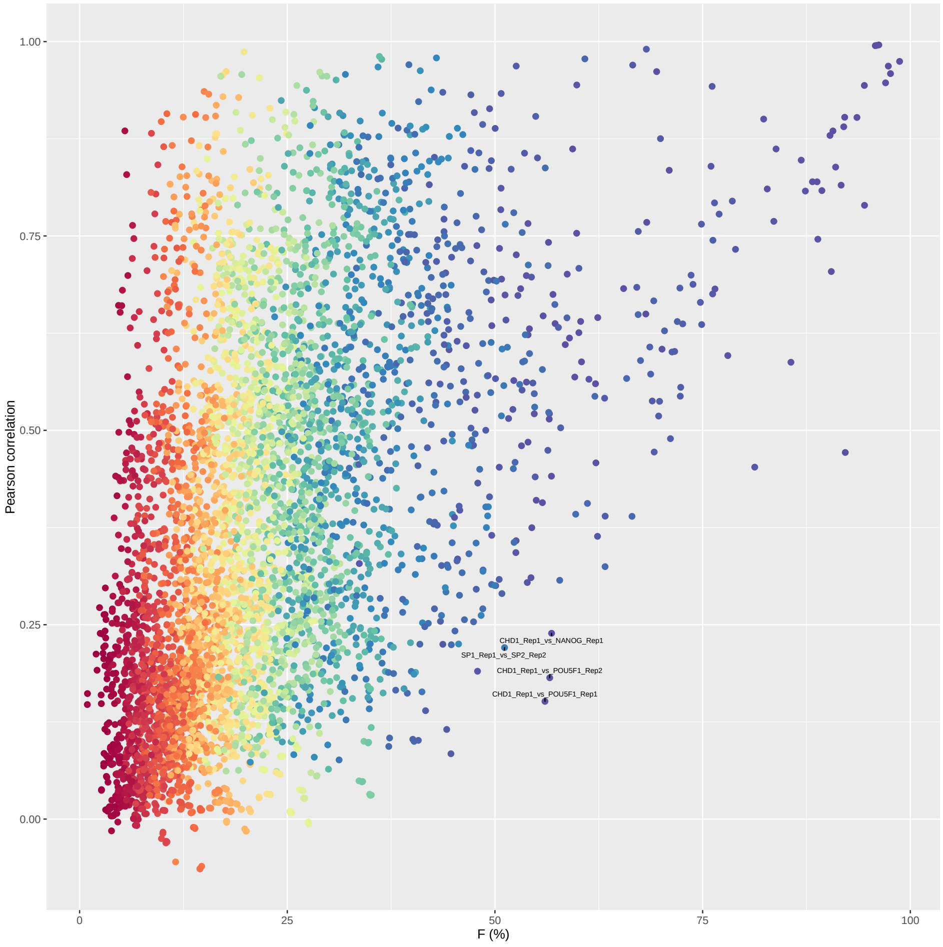

Supp. Figure 11. Pearson correlation coefficient versus F values for all combinations of interactions in H3K4me3 regions. Only comparisons with $F > 50\%$ and Pearson's $r < 0.25$ have been labelled in the plot.

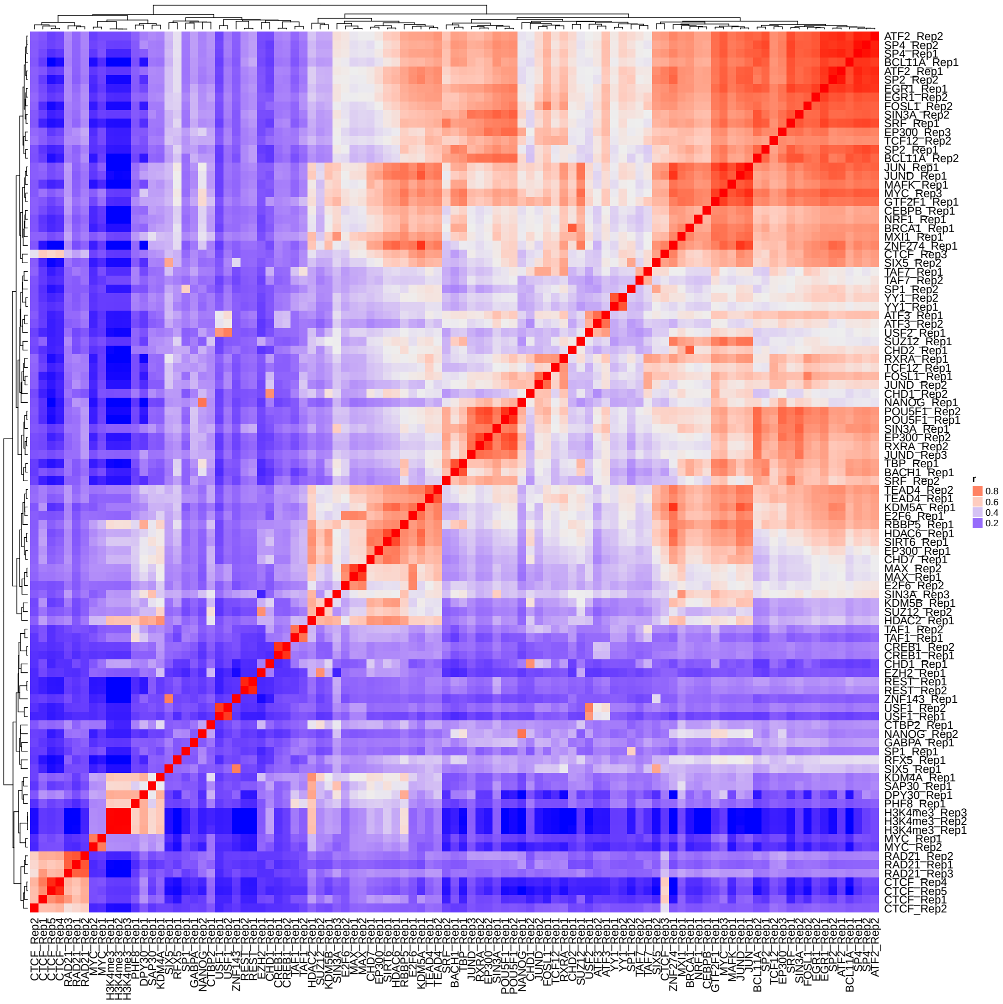

Supp. Figure 12. Heatmap for Pearson correlation coefficients r for all possible combinations of interactions in H3K4me3 peak regions.

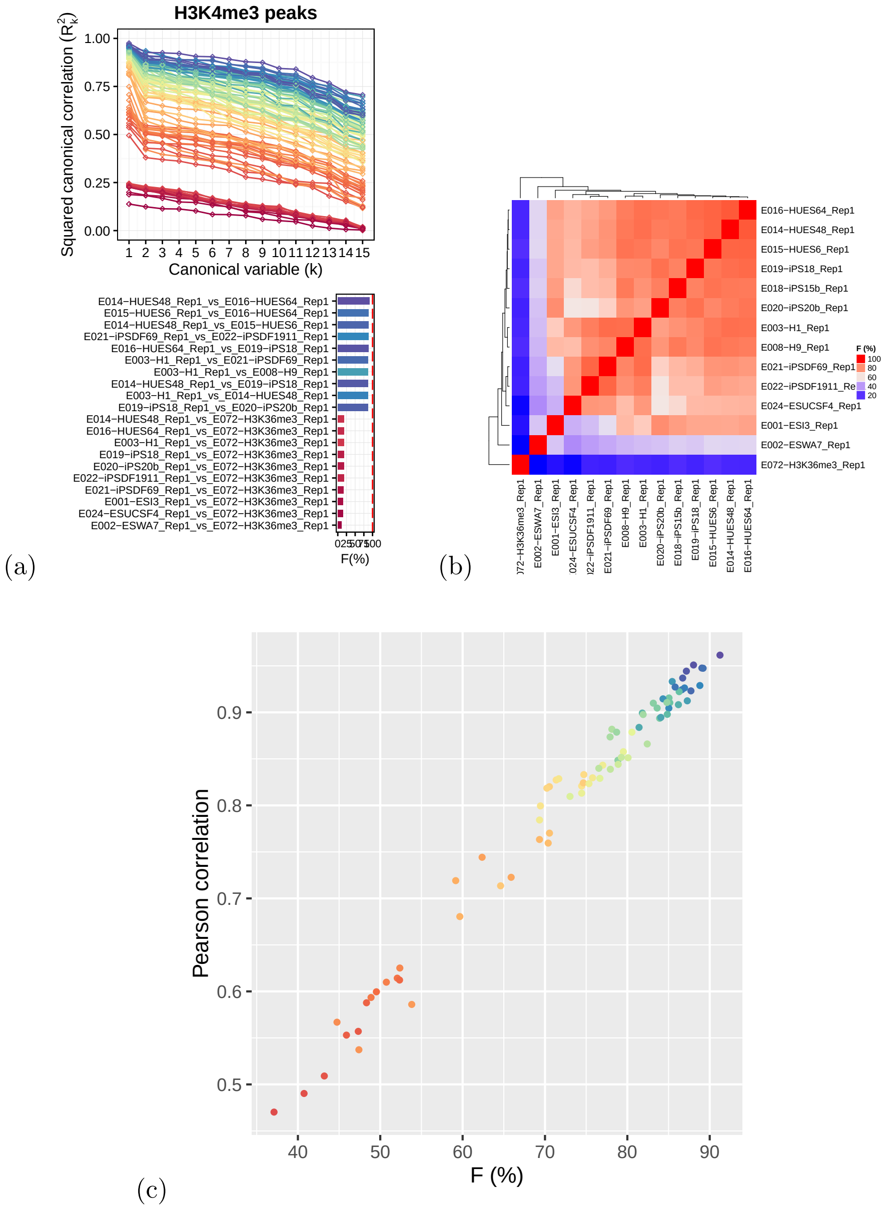

Supp. Figure 13. Assesment of covariance of H3K4me3 Roadmap Epigenomics Project samples in ES and iPS cells using functional canonical correlation analysis implemented in `fCCAC`. Chromatin mark H3K36me3 of brain inferior temporal lobe was randomly selected and introduced as a reference control. (a) Top: Squared canonical correlations for all interactions between datasets. Spectral colormap based in the value for $k = 1$. Bottom: First 10 and last 10 ranked interactions according to their percentage over maximum F. The red dashed line indicated perfect covariance. (b) Heatmap of F values for all possible combinations of interactions in H3K4me3 peak regions. (c) Pearson correlation coefficients versus F values for H3K4me3 peak regions.
